# A 47-year-old man with neuro-Sweet syndrome in association with Crohn's disease: a case report

**DOI:** 10.4076/1752-1947-3-8997

**Published:** 2009-09-10

**Authors:** Nadine Hiari, Colin Borland

**Affiliations:** 1 Hinchingbrooke Hospital, Huntingdon, PE29 6NT, UK

## Abstract

**Introduction:**

Sweet's syndrome is a multi-system inflammatory disorder characterised by painful skin lesions and aseptic neutrophilic infiltration of various organs. We describe a case of Sweet's syndrome with aseptic meningitis in association with Crohn's disease (neuro-Sweet syndrome). This association has never been previously reported.

**Case presentation:**

A 47-year-old Caucasian male with known Crohn's disease presented with headache, fever and skin lesions resembling erythema nodosum. The cerebrospinal fluid revealed leukocyte pleocytosis and dominant neutrophils, but cultures were negative. A skin biopsy revealed neutrophilic dermatosis compatible with Sweet's disease. The patient made a prompt recovery without the use of corticosteroids.

**Conclusion:**

Because of its multisystem nature, Sweet's syndrome may present diagnostic difficulty to specialists. Correct diagnosis by skin biopsy will prompt appropriate treatment.

## Introduction

Sweet's syndrome is a multi-system neutrophilic disease first described by Dr. Robert Douglas Sweet in 1964 [1] and is also known as "acute neutrophilic dermatosis" and predominantly affects women. The initial episode of classical Sweet's syndrome most frequently occurs between the ages of 30 and 60 often preceded by an upper respiratory infection. It may be associated with pregnancy, inflammatory bowel disease, malignancy (especially acute myeloid leukaemia) and drugs (especially granulocyte-colony stimulating factor) [2]. Su and Liu (1986) [3] proposed diagnostic criteria (major and minor) which were then modified in 1994 by Von den Driesch [4], who added elevated erythrocyte sedimentation rate (ESR) to the minor criteria. Su and Liu proposed two major and several minor criteria [3]. The major criteria are the following:

(i) Abrupt onset of tender or painful erythematous plaques or nodules occasionally with vesicles or pustules; (ii) Predominantly neutrophilic infiltration in the dermis without leukocytoclastic vasculitis and the minor criteria (a) Preceded by a nonspecific respiratory or GI tract infection or associated with inflammatory bowel disease, malignancy or pregnancy (b) Accompanied by periods of general malaise and fever >38°C; (iii) Abnormal laboratory values at presentation (three of four) ESR >20 mm/hr, positive C-reactive protein (CRP) value, greater than 8 × 10^9^ leucocytes/L, differential white blood count of 70% neutrophils or greater. For a definitive diagnosis both major and two minor criteria must be met.

Classical Sweet's syndrome is characterised by fever associated with typically tender erythematous skin lesions (papules, nodules or plaques). The skin lesions are commonly assymetrical and are most frequently found on the upper extremities, face and neck. The main pathological finding is of a diffuse infiltrate consisting predominantly of mature neutrophils located in the upper dermis. In addition, papillary oedema is characteristically present. Fragmented neutrophil nuclei, swollen endothelial cells and dilated small blood vessels may occasionally be present. The overlying epidermis is normal and changes of "primary" leukocytoclastic vasculitis are usually absent. Aseptic neutrophilic inflammation may also be found in other sites such as bones, intestines, liver, aorta, lungs and muscles [2].

A closely-related syndrome is neuro-Sweet disease in which in addition to the skin, parts of the central nervous system (CNS) are affected by an aseptic neutrophilic inflammation. Again, other organs including liver and kidneys may be involved. Hisanaga *et al*. have proposed diagnostic criteria for neuro-Sweet disease [5].

We describe a case of neuro-Sweet disease in a patient with Crohn's disease, an association not previously reported.

## Case presentation

A 47-year-old Caucasian male with a 10-year history of Crohn's disease presented with a 5-day history of fever, night sweats, rash over both legs and headache. The headache was localised at the left temporal area and associated with nausea, lethargy, general malaise, fever, night sweats and rigors but not photophobia. There was no history of previous upper respiratory tract infection. The medical history of the patient did not indicate anything relevant except Crohn's disease which was diagnosed in 1998. This was treated with a partial colectomy in 1999 and a total colectomy in 2006. He did not take any regular medication and reported no drug allergies.

**Figure 1:**
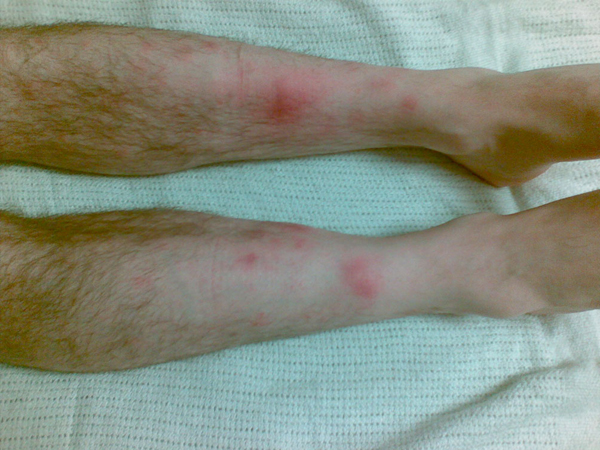
**Both legs**. Appearance of the anterior aspect of both legs at hospital presentation that is, 5 days after symptom onset.

**Figure 2:**
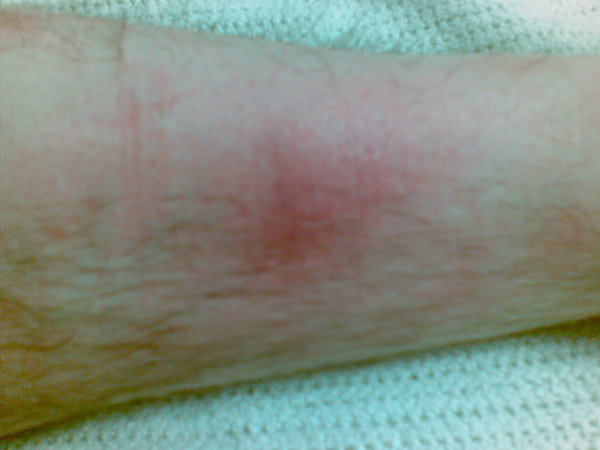
**Left leg**. Close-up appearance of the anterior aspect of the left leg at hospital presentation that is, 5 days after symptom onset.

**Figure 3:**
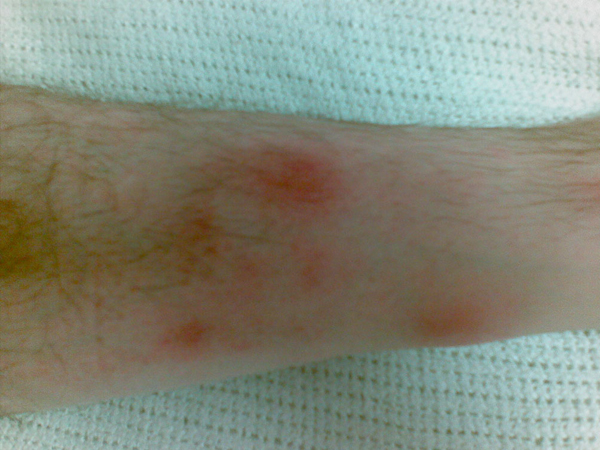
**Right leg**. Close-up appearance of the anterior aspect of the right leg at hospital presentation that is, 5 days after symptom onset.

On initial physical examination upon hospital admission, the patient was afebrile (tympanic temperature 36.5°C). His skin revealed lesions that were purple-red, non-pruritic, tender nodules measuring 1-2 cm in diameter. They were asymmetrically distributed over both shins and inner knee areas resembling eryhthema nodosum (Figures 1-3). Neurological examination was unremarkable. He had no neck stiffness.

Laboratory examination revealed a white cell count of 11.5 × 10^9^/L with neutrophilic leukocytosis (79%). CRP was raised (234 mg/l). Both kidney and liver functions were normal.

The next morning, the headache worsened and the patient began experiencing neck stiffness. On examination, the patient had a fever with temperature of 39.4°C. Mild neck stiffness was elicited. He did not have focal neurology. Further nodules had appeared on his shins.

A computed tomography (CT) head scan was normal. We proceeded to a lumbar puncture. This showed clear fluid, an opening pressure of 36 cm H_2_O, no xanthochromia, total cells 366, polymorphs 294, lymphocytes 72, red blood cells <1, cerebrospinal fluid culture negative (also negative for acid-fast bacilli) and meningococcal polymerase chain reaction negative. Since these results initially suggested bacterial meningitis, 2 g of intravenous ceftriaxone twice daily was started. In addition, the patient received ibuprofen 400 mg three times daily and paracetemol 1 g four times daily for analgesia.

On the third day of admission, further similar lesions appeared on both upper arms. Punch biopsies were taken from these lesions. Histology on the specimens revealed severe papillary edema. The dermis contained an infiltrate of neutrophils and nuclear dust was also present. These findings supported the clinical diagnosis of acute neutrophilic dermatosis (Sweet's disease.) On days 4-6 of admission, his symptoms started to improve. He was still having headaches and spiking temperatures of up to 38.9°C in the evenings but the skin lesions were settling with decreased tenderness and redness. By the seventh day, the patient was apyrexial, his headache had settled, and his CRP had fallen from 234 to 33. He was discharged from the hospital.

## Discussion

Neuro-Sweet disease (NSD) was first described as a distinct entity of aseptic encephalomeningitis by Hisanaga *et al.* in 1999 [5]. It affects males more frequently, with a male:female ratio of 1.3 : 1. The typical age at presentation is between 30 and 60 years old. There is no CNS site predilection and neurological sequelae are infrequent and generally mild. The encephalomeningitis may occur in any CNS region, resulting in various central symptoms including headache and altered level of consciousness. A fever of 38-40°C is observed. CSF studies usually reveal slightly elevated protein concentration and moderate pleocytosis. Increase in neutrophils, CRP and ESR are demonstrated in the peripheral blood.

Clinical diagnostic criteria for neuro-Sweet disease were proposed by Hisanaga *et al*. in 2005 [5]. These were:

(i) Neurologic features: highly systemic glucocorticoid responsive or sometimes spontaneously remitting, but frequently recurrent encephalitis or meningitis, usually accompanied with fever over 38°C. (ii) Dermatologic features: painful or tender, dull red erythematous plaques or nodules preferentially occurring on the face, neck, upper limbs and upper part of trunk; predominantly neutrophilic infiltration of the dermis, spared epidermis and absence of leukocytoclastic vasculitis. (iii) Other features: Absence of cutaneous vasculitis and thrombosis which are seen in BehÇet's disease, absence of typical uveitis, which is seen in BehÇet's disease. (iv) HLA Association: HLA-Cw1 or B54 positive, HLA-B51 negative. For probable neuro-Sweet disease 1, 2, 3 are required and possible neuro-Sweet disease either 2 or 4 and one more item.

Neuro-Sweet disease skin lesions, if untreated, can remain for weeks, even months. However, without any therapeutic intervention, the dermatosis-related symptoms and lesions eventually resolve in some patients with classical Sweet's syndrome. The encephalomeningitis may remit spontaneously or with systemic corticosteroid therapy (prednisolone 1 mg/kg/day) as first line of pharmacological management, which is the therapeutic mainstay for Sweet's and neuro-Sweet disease syndrome. Alternative first-line systemic treatments are potassium iodide and colchicine. Second-line agents include indomethacin, cyclosporine and dapsone.

## Conclusion

This is a case of aseptic meningitis secondary to neuro-Sweet disease in a patient with Crohn's disease. The association of Crohn's disease with Sweet's syndrome was previously described in the literature. This is the first case, to our knowledge, of the association of neuro-Sweet disease with Crohn's disease.

Because of its multisystem nature, Sweet's syndrome may present to a range of specialists. Typically patients with meningitis or encephalitis will present on the unselected medical intake and may be treated with antibacterials or acyclovir. As in our case, the skin lesions, to the general physician, may resemble erythema nodosum. Fever is unusual in erythema nodosum. Patients under the care of a specialist gastroenterological service may show their skin lesions first to specialist doctors or nurses. Correct diagnosis by dermatological referral and a skin biopsy, if necessary, will prompt appropriate treatment.

### Patient's perspective

I first noticed aches around my knees but ignored it because I had recently performed a series of leg stretch exercises; I thought I had overdone my stretching. Over the next few days my aches continued, especially noticing the pain when climbing stairs. I had also felt slightly lethargic, which I thought was because I was near due my B12 shot, over these few days and often irritable. I also had a constant headache that seldom relented. My wife convinced me to see visit my GP because she noticed red knots on my knees. I agreed because I wanted rid of the headaches, which have now been around for about a week.

Today - some 15 weeks later - I am very conscious of a repeat occurrence (for example, whenever I get a headache or muscle ache, I monitor them).

## Abbreviations

CNS: central nervous system; ESR: erythrocyte sedimentation rate; HLA: human leucocyte antigen; NSD: neuro-Sweet disease.

## Consent

Written informed consent was obtained from the patient for publication of this case report and accompanying images. A copy of the written consent is available for review by the Editor-in-Chief of this journal.

## Competing interests

The authors declare that they have no competing interests.

## Authors' contributions

NH wrote the report and reviewed the literature. The patient was admitted under the care of CB who critically reviewed and altered the format of the draft article to conform to the template for this journal.
